# Lactacystin: first-in-class proteasome inhibitor still excelling and an exemplar for future antibiotic research

**DOI:** 10.1038/s41429-019-0141-8

**Published:** 2019-02-12

**Authors:** Satoshi Ōmura, Andy Crump

**Affiliations:** Kitasato Institute for Life Sciences, Kitasato University, 5-9-1 Shirokane, Minato-ku, Tokyo, 108-8641 Japan

**Keywords:** Drug discovery and development, Drug discovery

## Abstract

Lactacystin exemplifies the role that serendipity plays in drug discovery and why “finding things without actually looking for them” retains such a pivotal role in the search for the useful properties of chemicals. The first proteasome inhibitor discovered, lactacystin stimulated new possibilities in cancer control. New and innovative uses are regularly being found for lactacystin, including as a model to study dementia, while new formulations and delivery systems may facilitate its use clinically as an anticancer agent. All this provides yet more evidence that we need a comprehensive, collaborative and coordinated programme to fully investigate all new and existing chemical compounds, especially those of microbial origin. We need to do so in order to avoid failing to detect and successfully exploit unsought yet potentially life-saving or extremely advantageous properties of microbial metabolites.

## Introduction

It has been a mere 100 years since the dawn of the modern era of microbial chemotherapy and the discovery of potent naturally-produced antibiotics. The discovery of penicillin in 1928 led to its development and clinical availability in 1942. Thereafter followed a Golden Age of antibiotics, with hundreds—some natural products, some synthesized—approved for use during the 1950s and 1960s. Yet over the following 2–3 decades, the number of new antibiotics discovered and entering into clinical use steadily decreased, leading to a current potentially catastrophic lack of effective products. In 2017, the WHO clearly identified the prevailing dire predicament in a report entitled ‘Antibacterial agents in Clinical Development’ which emphasised the urgent need for greater global investment in research and development (R&D) for antibiotics. Speaking at the launch of the report, the WHO Director-General concluded that R&D is imperative, “otherwise we will be forced back to a time when people feared common infections and risked their lives from minor surgery”.

However, not all is doom and gloom, as exemplified by Brad Spellberg, one of the authors of the 2004 Infectious Diseases Society of America (IDSA) report ‘Bad bugs, no drugs’ who drew attention to the possible answer to the problem; “given that the antibiotics we have available today were discovered as growth by-products of bacteria that we can culture, and that we’ve cultured less than 1% of the bacteria on our planet, there are many potential solutions out there”.

It has become patently obvious that microorganisms produce an immeasurable cornucopia of chemicals, including some which have already proved to be essential compounds for use in human medicine, agriculture, industrial processes and manufacturing. Unfortunately, to date, we have found only a minimal number of the vast array of constantly evolving microorganisms that exist in Nature. Those microorganisms produce virtually unlimited quantities of useful, often unique, molecules. The problem is that we have so far found literally only a meagre handful of those available. And evidence is accumulating rapidly, as demonstrated by the example of lactacystin, that when we do find some novel chemicals, we do not really know what they may be useful for or the variety of desirable properties they may possess which can be exploited to meet our ever-changing immediate as well as future needs.

A limited search image, restrictive technology, shortage of expertise and lack of resources are all elements combining to hinder researchers from appreciating the true scope of any microbial product. Personal experience in the history of the Kitasato Institute in Tokyo, one of the world’s premier facilities concentrating on natural products research, continues to reinforce this belief. Meanwhile, in industry, chemical companies are opening up their libraries of chemicals for new testing to determine any possible as-yet undetected properties that may be useful for combatting neglected or other diseases and conditions.

So what can the lesson of lactacystin tell us? Most notably, that scientists often find what they are looking for, but not what actually exists, particularly with respect to microorganisms and, by extension, the chemicals they can produce. If we are to find the new antibiotics so desperately needed and simply sustain our successes against all manner of threats to our survival existing in our ever-changing living environment, we need to proactively devise coordinated mechanisms to fully explore and discover what Nature produces. Cures for cancer, dementia, viral and bacterial diseases, etc., may be among many other highly beneficial tools and substances lying hidden or dormant on the shelves of chemical libraries around the world, as well as in the immeasurable panoply of microorganisms that we have not yet even identified, let alone investigated.

The evidence from work at the Kitasato Institute alone is quite compelling. The avermectins and ivermectin were discovered and developed because that was what was being looked for. The myriad and continually expanding properties, uses, potential and promise of ivermectin are slowly becoming manifest, some four decades after its discovery [1]. Likewise with staurosporine [2], and with lactacystin, as we will detail in this article.

Following the examples of the ‘first-in-class’ compounds ivermectin and staurosporine, the first-in-class naturally occurring lactacystin is another example of the global scientific community’s lack of a cohesive approach to fully comprehend what microbial compounds can offer to help improve the lives and welfare of the world’s population.

## Lactacystin discovery

In our experience, one-third of soil isolates examined produce antimicrobial substances. The key to observing and identifying these bioactive substances is the development of customised and effective screening systems. A practicable screening system requires the following: (i) the introduction of innovative mechanisms to reliably isolate, cultivate and identify microorganisms; (ii) the ability to assay a small quantity of secondary metabolite chemical easily and quickly; (iii) the use of appropriate biological material (such as bacteria, animal cells, enzymes) to facilitate detection of bioactivity. As part of our research programmes, we have developed several innovative screening systems, including a simple Physico-Chemical mechanism (making the use of colour changes in a reagent) and the use of animal cells (neuroblastoma cell lines, which have been used to test novel compounds for neurotoxic properties and associated mechanisms). Such transformed cell lines frequently exhibit morphological, developmental and signalling characteristics that are substantially different from the parental cell type.

**Fig. 1:**
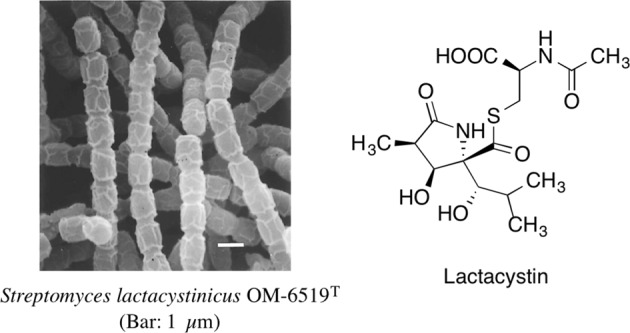
Structure of lactacystin and producing organism

**Fig. 2:**
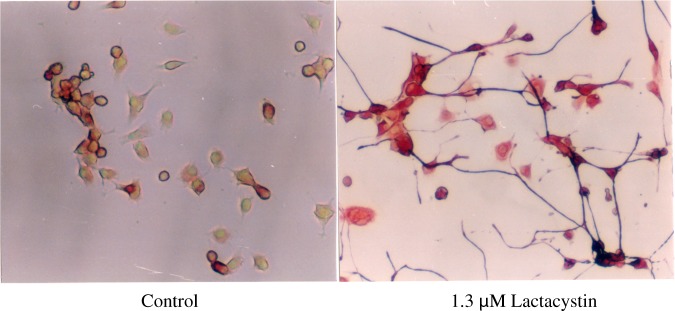
Immunofluorescence staining of 200 kDa neurofilaments in Neuro 2a cells

Lactacystin, first reported in 1991, is an organic compound naturally synthesized by soil-dwelling bacteria of the genus Streptomyces. It was discovered through our first foray into the use of animal cells in a screening system. Isolated from a culture broth of *Streptomyces lactacystinaeus* (Fig. 1), lactacystin was found to inhibit cellular growth and to be an inducer of neurite outgrowth in Neuro 2a cells, a murine neuroblastoma cell line (Fig. 2). The structure of lactacystin, elucidated by spectroscopic analyses, including NMR and X-ray crystallography, comprises a non-peptide skeleton consisting of two α-amino acids, N-acetylcysteine and a novel pyroglutamic acid derivative [3, 4]. The first total synthesis of lactacystin was accomplished in 1992, barely a year after its discovery [5].

Lactacystin was first isolated and identified at the Kitasato Institute at around the same time as the discovery of the proteasome in the late-1980s. The discovery of the proteasome, the central protease of what would later be termed the ubiquitin-proteasome system (UPS), rapidly followed by development of specific inhibitors of this enzyme complex, heralded a new era in biomedicine. Understanding of the central role of the proteasome complex in intracellular proteolysis, coupled with the fact that the proteasome is an essential factor in most, if not all, cellular processes, galvanized an intensive search for novel compounds possessing both increased selectivity towards the proteasome and better bioavailability for use clinically or in basic research.

Proteasomes are large multi-subunit proteases found in the cytosol (both free and attached to the endoplasmic reticulum) and in the nucleus of eukaryotic cells. Their presence and abundance reflects their central role in cellular protein processing [6]. A proteasome comprises two sub-complexes: a catalytic core particle (CP; also known as the 20S proteasome) and one or two terminal 19S regulatory particle(s) (RP) that serve as activators, with a molecular mass of approximately 700 kDa. The RP binds to one or both ends of the 20S proteasome to produce an enzymatically active unit. The RP recognizes ubiquitylated proteins and is thought to play a role in their unfolding and translocation into the interior of the 20S core. The sedimentation coefficient of a proteasome with one RP is 26S and the complex is usually referred to as the 26S proteasome, which is a 2.5-MDa multi-catalytic degradation component. If a pair of symmetrically disposed RPs are attached to both ends of the CP, the elongated 30S molecule is formed, which is probably the functional unit in the cell. The 20S proteasome consists of four heteroheptameric rings (two outer α rings and two inner β rings) with α1-7β1-7β1-7α1-7 stoichiometry. The 20S proteasome exposes catalytic threonine residues at the inner surface of the chamber formed by the two abutting β rings, with β1, β2, and β5 exerting caspase-like, trypsin-like, and chymotrypsin-like activities, respectively. How the complex structures of the 20S and 26S proteasomes are organized remains unclear. Proteins damaged by oxidation or intrinsically unstructured or unfolded proteins are directly degraded by the 20S proteasome. They are predominantly distributed in the nuclei of rapidly proliferating mammalian cells, indicating that they may contribute to cell proliferation. The balance between 20S and 26S proteasomes fluctuates in response to environmental conditions [7].

Proteasomes undertake metabolic energy-intensive selective, efficient and step-wise hydrolysis and degradation of intracellular proteins. They first integrate with ubiquitin, which polymerizes, thereby allowing for coordinated proteolysis. The 20S proteasome is omnipresent and vital in eukaryotic cells. Some prokaryotes, including many archaea and the bacterial order Actinomycetales, also contain various 20S proteasome homologues.

The proteasome is a key element of the UPS, which is responsible for over 80% of cellular protein degradation and which impacts virtually all vital cell functions [8]. It plays a pivotal role in controlling a diverse range of fundamental cellular activities by rapidly and unidirectionally catalyzing biological reactions. Ubiquitin polymerization is the signal for the degradation of target proteins. By lysing or degrading ephemeral regulatory or structurally-aberrant proteins, the UPS effectively governs almost all basic cellular processes, including the cell cycle, signal transduction, apoptosis, immune responses, metabolism, protein quality control and development, regulation of gene expression, DNA repair, and response to oxidative stress [7, 9]. The establishment and progression of infectious diseases is determined by interactions between pathogens and host cells, with pathogens disrupting signalling pathways in host cells to promote their own survival and replication, as well as to avoid host immune responses. A wide variety of human pathogens, including *Salmonella enterica*, *Listeria monocytogenes*, *Yersinia enterocolitica*, *Mycobacterium tuberculosis*, and *Legionella pneumophila*, manipulate host ubiquitination mechanisms to safeguard their presence and ability to function inside the host [10–12]. Consequently, disruption of proteasome activity is a prime causal factor in the progression of the pathology of several clinical disorders, including inflammation, neurodegeneration and cancer.

Proteasome inhibitors therefore possess immense potential for development into drugs to treat a wide range of diseases, including immunologic, inflammatory, metabolic and neurological disorders, viral diseases, muscular dystrophies and tuberculosis [13, 14]. The fundamental importance of cellular proteolytic degradation inside and the role of ubiquitin in proteolytic pathways was acknowledged by the bestowal of the 2004 Nobel Prize in Chemistry to Aaron Ciechanover, Avram Hershko and Irwin Rose “for the discovery of ubiquitin-mediated protein degradation [15].

Proteasome inhibitors can be synthetic or natural compounds and are commonly categorized according to their origin. The Actinobacteria naturally produce proteasome inhibitors such as lactacystin. To some extent, lactacystin was found because it was what was being sought and therefore tested for, based on rational evaluation of an increasing evidence base of the potential for targeting the proteasome. The naturally-occurring lactacystin was initially identified by its ability to inhibit cell cycle progression and induce neurite outgrowth in neuronal cell lines Chemists have produced synthetic versions of it but its five chiral centres make its synthesis far from simple.

**Fig. 3:**
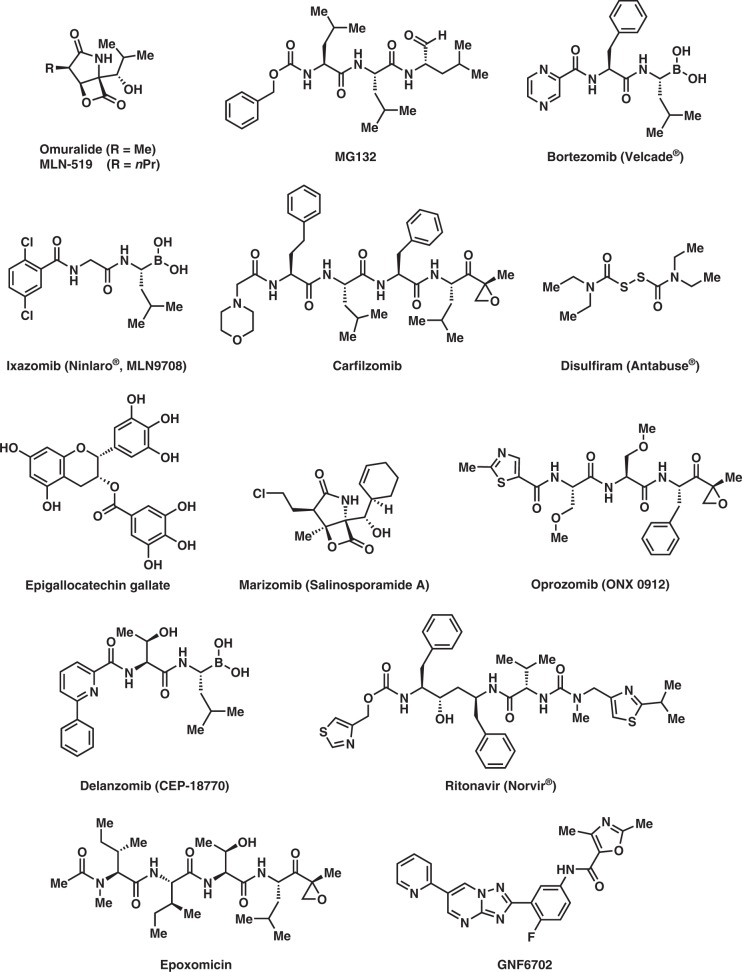
Structures of proteasome inhibitors

The first synthetic proteasome inhibitors discovered were peptide aldehydes which reversibly bind and block the proteasome, most lack specificity but not the frequently used inhibitor MG132 (Fig. 3). Lactacystin was the first natural non-peptidic proteasome inhibitor identified. It functions as a pro-drug in vivo, spontaneously generating the cell-permeable, biologically active clasto-lactacystin-β-lactone, also known as Omuralide (Fig. 3), with the concomitant elimination of N-acetylcysteine. Omuralide is bioactive but a far less stable compound than lactacystin [16–18].

Lactacystin binds and inhibits specific catalytic subunits of the proteasome which is responsible for the bulk of cellular proteolysis as well as proteolytic activation of certain protein substrates. Lactacystin is a specific, potent and irreversible proteasome inhibitor, with an IC_50_ value of 4.8 μM [16]. Consequently, it became the primary reagent for studying the role of the proteasome [19–21]. The molecule is a lactam, or cyclic amide and a number of methods to synthesize it have now been published, with more than 1645 citations for lactacystin appearing in PubMed as of 2018.

The proteasome comprises a 20S catalytic core plus additional subunits believed to be involved in the recognition and unfolding of ubiquitinated proteins; the composite structure having a sedimentation coefficient of 26S. Lactacystin binds certain catalytic subunits of the 20S proteasome. At least one of these subunits is modified by lactacystin on the side-chain hydroxyl of the amino-terminal threonine [16], which appears to function as the catalytic nucleophile in the proteolytic mechanism. Lactacystin inhibits the three well-characterized, distinct peptidase activities of the proteasome, chymotrypsin-like, trypsin-like and caspase-like, the first two irreversibly and all at different rates [16]. The β-lactone compound (Omuralide) inhibits each of the three activities 15–20 times faster than lactacystin, with the same order of effectiveness. Lactacystin also inhibits peptide hydrolysis by the larger 26S complex and inhibits ubiquitin/proteasome-mediated degradation of short- and long-lived proteins in the cell [22]. This small molecule, together with epoxomicin, are the only two compounds known to inhibit the proteasome specifically without inhibiting any other protease in vitro. Significantly, it does not inhibit lysosomal protein degradation in the cell. This is in contrast to other commonly used proteasome inhibitors, such as peptide aldehydes and 3,4-dichloroisocoumarin, which inhibit a wide range of proteases.

Lactacystin, the first non-peptidic proteasome inhibitor discovered, quickly became widely exploited as a research tool in biochemistry and cell biology. It was licensed to Myogenics/Proscript, which was acquired by Millennium Pharmaceuticals, now part of Takeda Pharmaceuticals.

## Mode of action

Proteasomes recognize, unfold, and digest protein substrates that have been marked for degradation by the attachment of a ubiquitin moiety [23]. Lactacystin binds specifically to the 20S proteasome β-5 subunit [24, 25] and inhibits the hydrolysis of intracellular peptides normally carried out by the 26S complex and by the UPS [22].

Lactacystin’s target protein is the β-5/PRE2 in the complex enzyme. It covalently modifies the amino-terminal threonine of catalytic β-subunits, particularly that responsible for the proteasome’s chymotrypsin-like activity. This discovery helped to establish the proteasome as a mechanistically novel class of protease: an amino-terminal threonine protease. Lactacystin blocks multiple activities of the proteasome through modification of all the major catalytically active β-subunits [26]. It acts by spontaneous lactonization between its thioester and the hydroxyl group at carbon C6 to form the highly reactive Omuralide. In contrast to synthetic peptide aldehydes, structurally distinct natural inhibitors such as lactacystin covalently bind to proteasome subunits, irreversibly blocking all proteolytic activity [27]. In aqueous environments, lactacystin undergoes spontaneous hydrolysis to clasto-lactacystin dihydroxic acid and N-acetylcysteine, along with the intermediary clasto-lactacystin-β-lactone (Omuralide) [28]. Omuralide forms an ester-linked adduct with the amino-terminal threonine of the mammalian 20S proteasome β-5 subunit. Through covalent attachment, Omuralide potently inhibits all three peptidase activities of the 20S proteasome. Lactacystin (via the intermediary Omuralide) is highly specific for the proteasome and does not inhibit serine and cysteine proteases [19] or lysosomal protein degradation [26]. Some of the first synthetic proteasome inhibitors were peptide aldehydes that act as substrate analogues and potent transition-state inhibitors, primarily of the chymotrypsin-like activity of the 20S proteasome [29]. This lactone species is the primary agent responsible for alkylation of the proteasome’s active site. Lactonization is necessary for inhibition of the proteasome in intact cells. Unlike lactacystin, Omuralide is cell permeable and can enter cells, where it interacts with the 20S proteasome. The lack of structural resemblance of lactacystin to synthetic peptide-based protease inhibitors bestows its high specificity, leading many to adopt it as the ‘Gold standard’ by which to judge specificity of proteasome inhibitors [28, 30]. Consequently, Omuralide is now extensively used and is commercially available.

## Anticancer properties

Because the UPS plays an essential role in regulating homeostatic and various cellular events, including those involved in tumorigenesis, targeting it is a rational approach with respect to human cancer treatment [31–34]. Proteasome inhibition leads to the accumulation of pro-apoptotic proteins in tumorigenic cells but not in normal tissue [35, 36]. Lactacystin was the first natural product proteasome inhibitor found and the first-in-class compound quickly became extremely useful in cancer therapy and research. It proved to be not only a highly specific anticancer agent itself but also an excellent lead compound for development of other anticancer products.

Lactacystin inhibits basic cell proliferation, its antineoplastic activity being dependent on inhibition of the UPS, which controls cellular protein turnover [6]. In Neuro-2a cells, lactacystin induces neurite outgrowth, a transient rise in cAMP levels and bipolar morphology of the cells. Moreover, lactacystin exhibits selective proteasome inhibition in cells undergoing neoplastic transformation [37–39]. It also potentiates and enhances the impact of chemotherapeutical agents [40] in addition to sensitizing cell lines to both radiotherapy [41] and chemotherapy [42, 43], and may affect tumour growth by inhibiting angiogenesis [44].

Lactacystin blocks cell cycle progression in MG-63 human osteosarcoma cells. It also displays therapeutic effects on glioma cells, in rat C6 glioma inhibiting cell growth with an IC_50_ value of about 10 μM. In a mouse glioma xenograft model, administration of lactacystin at a dose of 1–5 μg/20 g body weight significantly reduced tumour size. Lactacystin has also been shown to trigger apoptotic cell death in several human glioma cell lines [45–48]. Recently published clinical studies have shown that proteasome inhibitors are effective in the treatment of both solid and haematological malignancies. Preclinical studies in cell culture and animal models have identified the anti-tumour potential of proteasome inhibitors against many types of cancer cells including melanoma [49–54]. As a result, a multitude of molecules that target the proteasome have been identified or rationally designed.

Omuralide is attracting increasing attention because it is a low-molecular-weight, potent and selective proteasome inhibitor. It is nearly optimal for inactivation of the 20S proteasome, with simple replacement of the C-7 methyl group by longer aliphatic chains enhancing its inhibitory potency 2- to 3-fold. An analogue with a C-7 n-propyl substituent, PS-519 (also known as MLN-519 (Fig. 3) produced by Millennium Pharmaceuticals Inc. (formerly LeukoSite Inc.)) has been evaluated in Phase I clinical trials for acute stroke. Millennium and PAION GmbH are also investigating MLN-519 for treatment of inflammatory diseases and it is in advanced clinical trials against acute stroke and myocardial infarction [55].

Systemic administration of lactacystin for the treatment of brain tumours would require very high doses to effect penetration through the blood brain barrier (BBB), which would cause severe toxicity. This has precluded lactacystin from being used clinically. Nevertheless, targeted delivery of drugs using controlled-release mechanisms is now a safe alternative for administration of potentially toxic chemotherapeutical agents, including for malignant brain tumours. Sustained-release polymers circumvent the BBB, thereby allowing targeted but local high concentrations of the therapeutic compound while preventing systemic toxicities [56].

The success of the proteasome inhibitor bortezomib, originally synthesized in 1995 and marketed as Velcade^®^ in 2003 for the treatment of multiple myeloma, and clinical trials to evaluate the effect of several other proteasome inhibitors in various human pathologies [57, 58], emphasize the potential of proteasome inhibitors for improving human health and saving lives.

### Multiple myeloma

Phase I and II clinical trials established that bortezomib (boronated MG132) can be administered with acceptable and manageable toxicity and that it was efficacious in the treatment of various malignancies, especially multiple myeloma as well as non-small cell lung carcinoma and non-Hodgkin’s lymphoma, among others [59–62].

Bortezomib, developed by Millennium Pharmaceuticals and marketed as Velcade^®^, was the first proteasome inhibitor to enter clinical use. It was approved by the US Food and Drug Administration (FDA) as the first-in-class proteasome inhibitor, for use as a third-line therapy in multiple myeloma, with the European Union registering it a year later [63]. In 2003, bortezomib was approved for multiple myeloma as a second-line treatment on its own and as a first-line therapy (in combination with an alkylating agent and a corticosteroid). Furthermore, bortezomib has also been approved as a second-line therapy for mantle cell lymphoma [64, 65].

Multiple myeloma is associated with increased proteasome-derived peptide levels in blood serum that subside to normal levels in response to chemotherapy [66]. Three proteasome inhibitors are now approved for use in multiple myeloma (Fig. 3);

1. Bortezomib (Velcade^®^) was approved in 2003 (7 years after original synthesis). Its boron atom binds the catalytic site of the 26S proteasome [67].

2. Carfilzomib (Kyprolis^®^) was approved by the FDA for relapsed and refractory multiple myeloma in 2012 [68]. It irreversibly binds to and inhibits the chymotrypsin-like activity of the 20S proteasome.

3. Ixazomib (Ninlaro^®^) was approved by the FDA in 2015 for use, in combination with lenalidomide and dexamethasone, for the treatment of multiple myeloma after at least one prior therapy. It is the first orally-available proteasome inhibitor [69].

Animal studies have demonstrated that bortezomib may also have clinically significant effects in pancreatic cancer [70, 71]. Clinical results also demonstrate the value of a proteasome inhibitor combined with chemotherapy for B-cell acute lymphoblastic leukemia [72]. Proteasome inhibitors can also kill some types of cultured leukemia cells that are resistant to glucocorticoids [73].

Several other proteasome inhibitors (Fig. 3) are in drug development stages or are undergoing clinical trials and testing, including ONX-0912 (oprozomib) for Waldenstrom’s macroglobulinaemia (a type of non-Hodgkin lymphoma (NHL)) and multiple myeloma; CEP-18770 (delanzomib) for multiple myeloma; Salinosporamide A (marizomib) for glioma; disulfiram (Antabuse^®^) for alcoholism, and Epigallocatechin-3-gallate, as a supplement for lowering cholesterol,

In addition, the molecule ritonavir (Fig. 3), marketed as Norvir^®^, was developed as a protease inhibitor and used to target HIV infection. However, it has been shown to inhibit proteasomes as well as free proteases, the chymotrypsin-like activity of the proteasome being inhibited by ritonavir whereas the trypsin-like activity is somewhat enhanced [74]. Animal model research suggests that ritonavir may have inhibitory effects on the growth of glioma cells [75].

### Skin cancer

The incidence of malignant melanoma, the most aggressive skin cancer, is increasing constantly. It is exceptionally difficult to treat since it metastasizes early and is often resistant to conventional chemotherapy and radiotherapy. Despite new targeted therapies, the prognosis for patients with metastatic disease remains poor. There is therefore an urgent need for new drugs or new combination therapy, and UPS inhibitors are potentially valuable antineoplastic agents in this respect. The proteasome is an enticing target for chemical intervention due to its essential role in cellular physiology. Proteasome-mediated cyclin degradation is required for initiation of mitosis, and this suggests yet another role for inhibitors as anticancer agents. Both lactacystin and epoxomicin (Fig. 3) are covalent, specific inhibitors of the proteasome that have been chemically synthesized, and therefore can be easily converted into a labelled form. Although they remain two of the most useful inhibitors currently available, the relatively high cost of obtaining them from commercial sources limits their frequent use [76].

### Gastric cancer

Research has shown that lactacystin has diversified killing effects on gastric cancer cells. The mechanism involved may be related to the induction of apoptosis by downregulation of nuclear factor kappa B viability [77].

### Anticancer drug potentiation

Lactacystin has been found to be a valuable enhancer of existing anticancer treatment. It is a good potentiator of anticancer drugs, such as cisplatin, as well as when given with anticancer radiotherapy. Cisplatin is a widely used chemotherapy drug that is cytotoxic through affecting both nuclear and cytosolic pathways. It is commonly used in anticancer treatment despite its known adverse side effects and increasingly spreading drug resistance, meaning that novel methods for combination therapy with cisplatin will be required to overcome the limitations of cisplatin monotherapy. Lactacystin, which is known to produce antitumour effects, has been shown to work well in this respect. Like lactacystin, cisplatin inhibits proteasome activity in vitro and it also induces a dose-dependent inhibition of the three enzymatic activities of the proteasome (i.e., the chymotrypsin-like, trypsin-like activity and caspase-like activity). Simultaneous administration of lactacystin and cisplatin enhances the cytotoxicity of cisplatin alone [78]. In HeLa human cervical cancer (HCC) cells, cisplatin treatment inhibits cell growth and induces cell apoptosis. HeLa cell exposure to cisplatin-induced endoplasmic reticulum (ER) stress-associated apoptosis, coupled with lactacystin treatment, resulted in elevated levels of cell apoptosis and activation of caspase-3. Specifically, lactacystin treatment increased the cisplatin-induced expression of PDI, GRP78, CHOP and cleaved both caspase-4 and caspase-3. Together, these data indicate that lactacystin enhances cisplatin cytotoxicity by increasing ER stress-associated apoptosis [79].

### Neurodegenerative disorder

Neurotrophic factors (NTF) are known to be essential for the survival and functional maintenance of nerve cells [80–82]. The lack or decline of NTF is considered to cause dysfunction of the nervous system, resulting in various nerve diseases, including dementia, such as Alzheimer’s disease (AD). Degradation of old and damaged proteins, through the proteasome and autophagy-lysosome systems, decreases with age, thus negatively altering the vital balance between protein synthesis and protein clearance, impacting both proteasome structure and function. This altered balance influences age-related neurodegenerative disorders, likely increasing the risk of protein accumulation disorders, including AD, since deposition of amyloid and tau proteins develop long before the onset of cognitive symptoms [83–86].

At the turn of the millennium, the world began to pay greater attention to mental health issues and neurodegenerative disorders. The incidence of conditions such as dementia (including AD) and Parkinson’s disease (PD) became matters of increasing concern, stimulating a burgeoning research effort to devise products, tools and techniques to prevent, slow down progression of or treat them. The pathological indicators of PD are depigmentation of the substantia nigra pars compacta (SNc) due to the progressive loss of nigral dopaminergic neurons and the formation of intracellular inclusions, termed Lewy bodies, in surviving neurons [87]. The proteinaceous Lewy bodies are used as an indicator for PD. They contain insoluble α-synuclein (aSyn) and many other ubiquitinated proteins, suggesting a role for protein degradation system failure in PD pathogenesis. Protein accumulation in large insoluble cytoplasmic aggregates is thought to result, partially if not fully, from disruption of intracellular protein degradation pathways. Neurons that produce nigral dopamine (DA) are rendered dysfunctional and are progressively lost during a lengthy period as the disease progresses. Gradual degeneration of the nigrostriatal DA-ergic pathway leads to the manifestation of motor-related symptoms, such as resting tremor, difficulty in initiating movements (akinesia), slowness of movements (bradykinesia), muscular rigidity and loss of balance. An increasing body of evidence also indicates that proteasome function is impaired in the substantia nigra of PD patients [88].

The use of lactacystin therefore provides an excellent animal model to facilitate and expedite PD research. Following the discovery of structural and functional deficits in the proteasome system in patients with PD, the proteasome inhibition model was introduced during the early 2000s. It uses proteasome inhibitors to induce disturbances in proteostasis and trigger DA neurodegeneration [89]. The proteasome-inhibition PD model is singular because it induces nigral DA neurodegeneration via a distinct, but highly relevant, action mechanism. The model mimics the accumulation and aggregation of endogenous aSyn, something that was difficult to accomplish in previous non-genetic PD models. As a consequence of treatment with proteasome inhibitors, rodents display motor impairment which is responsive to DA-ergic drugs [90].

Lactacystin injected into rodent brains mimics several of the symptoms of PD. Administration into the SNc of rats leads to nigrostriatal DA neurodegeneration and motor impairment [91–97]. The lactacystin-induced DA cell death is probably apoptotic in nature, as indicated by ultrastructural assessment using electron microscopy [98]. Microinjection of lactacystin above the substantia nigra pars compacta in C57Bl/6 mice induces a PD-like motor phenotype 5–7 days after injection in both young and adult mice. This was associated with widespread neuroinflammation (based on glial cell markers), aSyn accumulation in the substantia nigra, striatal dopamine reduction, and loss of dopaminergic cell bodies in the substantia nigra and terminals in the striatum [99]. It has also been suggested that the determinants of cell death involve diverse cellular pathways related to production of reactive oxygen species, disturbances in mitochondrial function, induction of endoplasmic reticulum (ER) stress, and cytoplasmic accumulation of p53, iron and aSyn. In essence, the lactacystin-proteasome inhibition model has emerged as a new PD model that should help uncover vital evidence to help understand the complex pathogenesis of PD, as well as offer a means to investigate neuroprotective therapies.

## Other potential uses

### Neglected tropical diseases (NTDs)

Chagas disease, leishmaniasis and sleeping sickness afflict around 20 million people worldwide and lead to more than 50,000 deaths annually [100]. The diseases are caused by infection with the kinetoplastid parasites *Trypanosoma cruzi*, *Leishmania* and *Trypanosoma brucei*, respectively. These parasites have similar biology and genomic sequence, suggesting that all three could be tackled with drugs that target a conserved parasite target [101]. No such molecular targets or broad-spectrum drugs have been identified to date, although a selective inhibitor of the kinetoplastid proteasome has been reported. GNF6702 (Fig. 3) is a broad-spectrum antiprotozoal drug created in the Genomics Institute of the Novartis Research Foundation in 2013. It acts as a non-competitive proteasome inhibitor, effective against infection with any of the three protozoal parasites in mice but displaying minimal toxicity in mammalian cells.

It displays unprecedented in vivo efficacy, clearing parasites from mice in all three models of infection. GNF6702 inhibits the kinetoplastid proteasome through a non-competitive mechanism, does not inhibit the mammalian proteasome or growth of mammalian cells, and is well tolerated in mice. Genetic and chemical validation of the parasite proteasome as a promising therapeutic target for treatment of kinetoplastid infections, emphasizes the possibility of developing a single class of drugs for these three neglected diseases which have so far proved relatively hard to combat with all available drugs [102].

### Malaria

Proteasome inhibitors have been shown to be toxic for the malaria parasite, *Plasmodium falciparum*, at all stages of its life cycle [103–106]. Most compounds that have been tested against the parasite also inhibit the mammalian proteasome, resulting in toxicity that precludes their use as therapeutic agents [107]. Therefore, better definition of the substrate specificity and structural properties of the Plasmodium proteasome could enable the development of compounds with sufficient selectivity to allow their use as antimalarial agents. Design of inhibitors based on amino-acid preferences specific to the parasite proteasome found that they preferentially inhibit the β2-subunit. Interestingly, specific substrate assays proved that the chymotrypsin-like activity of the malarial proteasome was strongly inhibited by GNF6702, while mutant strains were far less susceptible.

Using cryo-electron microscopy and single-particle analysis, the *P. falciparum* 20S proteasome bound to the inhibitor was found to give a resolution of 3.6 Å. This reveals an unusually open *P. falciparum* β2 active site, providing valuable information about active-site architecture that can be used to further refine inhibitor design. Furthermore, consistent with the recent finding that the proteasome is important for stress pathways associated with resistance of artemisinin-family antimalarials, growth inhibition synergism with low doses of this β2-selective inhibitor in artemisinin-sensitive and artemisinin-resistant parasites has been observed. A parasite-selective inhibitor could be used to attenuate parasite growth in vivo without appreciable toxicity to the host. Consequently, the Plasmodium proteasome appears to be a chemically tractable target that could be exploited [108].

### Japanese encephalitis

Proteasome inhibitors are also now thought to be of potential use in tackling Japanese Encephalitis (JE), a viral disease that is the leading cause of vaccine-preventable encephalitis in Asia and the western Pacific. Human JE infections are often asymptomatic or result in only mild symptoms but a small percentage of those infected develop inflammation of the brain (encephalitis), with symptoms including sudden headache, high fever, disorientation, coma, tremors and convulsions. About 25% of cases are fatal. The host–virus interaction during the cellular entry of the JE virus remains poorly understood. The UPS, mediates various cellular processes, including endocytosis and signal transduction, which may be involved in the entry of virus. The proteasome inhibitors, lactacystin and MG132, both hindered successful entry of the JE virus by interfering with viral intracellular movement at the point between the virus crossing the cell membrane and uncoating, disrupting initial translation of the viral genome as a result [109].

### Hypertension

Lactacystin interferes with several factors involved in heart remodelling. Lactacystin treatment increases systolic blood pressure and induces fibrosis of the left ventricle (LV) in rats. Chronic administration of lactacystin represents a novel model of hypertension with collagenous rebuilding of the LV, convenient for testing antihypertensive drugs or agents exerting a cardiovascular benefit over and above blood pressure reduction [110].

### Oxidative stress

Proteasome inhibitors may induce endoplasmic reticulum (ER) stress and oxidative stress, disrupt signalling pathways, as well as activating apoptosis in several cancer cells. They also increase glutathione (GSH) synthesis and protect cells from oxidative stress. Lactacystin treatment increases reactive oxygen species (ROS) and GSH levels in HT-29 colorectal cancer cells. This suggests that oxidative stress arising as a result of proteasomal inhibition causes an elevation of cellular GSH levels, due to increased synthesis of GSH and uptake of cystine/cysteine. Following addition of lactacystin, enhanced expression of antioxidant components involved in GSH homeostasis is p38 MAPK-dependent, but Nrf2-independent, resulting in increased GSH synthesis capacity [111].

### Autoimmune disease

Proteasome inhibitors have also shown promise in treating autoimmune diseases in animal models. Studies in mice bearing human skin grafts found a reduction in the size of psoriasis lesions after treatment with a proteasome inhibitor [112]. Inhibitors also show positive effects in rodent models of asthma and could become a novel antiasthma intervention [113].

### Circadian rhythm

Most proteins, especially those that are short-lived, are degraded by the proteasome. Many physiological processes such as sleep/waking cycles, heart rate, blood pressure, and metabolism fluctuate in a daily cycle to maintain homeostasis and good health. These processes are influenced by or regulated by the circadian clock (CC), a biological mechanism that controls a range of physiological processes in many organisms based on an approximate 24 h cycle. Some proteins must be manipulated in a very precise and timely fashion and this also applies to components of the CC itself. Poorly controlled degradation of two circadian clock proteins (cryptochromes) in the mouse causes their accumulation throughout the day. Their presence at incorrect time points disrupts the expression of other CC-integral proteins and as a result, the length of the circadian cycle is extended [114].

### Bone disease

The UPS is critical in regulating the balance between bone formation and bone resorption. Proteasome inhibitors influence the activity of mature osteoblasts and osteoclasts, but also modulate the differentiation of precursor cells into osteoblasts. Preclinical studies show that melatonin also influences bone metabolism by stimulating bone growth and inhibiting osteoclast activity. These actions of melatonin could be interpreted as being mediated by the ubiquitin ligases SCF(B-TrCP) and Keap-Cul3-Rbx, or as an inhibitory effect on proteasomes. It may be that lactacystin or other proteasome inhibitors has a role to play in tackling diseases of the bone [115].

## New delivery systems

Even though lactacystin is known to induce apoptosis of cancer and other cells in vitro, its systemic toxicity prevents its clinical use. To bypass this problem, a new system of delivering lactacystin has been developed using biodegradable polymers which even allows it be effective intratumorally [116]. The efficacy of lactacystin incorporated into controlled-release polymers for treating experimental gliomas was evaluated against 9L-gliosarcoma and F98-glioma cell lines, treated with lactacystin (10–100 μ/ml) for 72 h in vitro. Following intracranial implantation in rats, the polymers released lactacystin for 21 days, with no observed local or systemic toxicity. Researchers concluded that lactacystin exhibits potent cytotoxic activity against 9L and F98 in vitro, and that it can be efficiently incorporated and delivered, using controlled-release polymers and at the required concentrations, the lactacystin polymers proving safe for CNS delivery and capable of prolonging survival in the 9L model [44].

## Concluding remarks

In our experience, in the past, new chemicals were discovered using common or unique assay systems with a distinct target or product in mind. Consequently, the full spectrum of the bioactivity possessed by many compounds may well have been overlooked because ‘what was being looked for had been found’. This certainly appears to be the case for ivermectin, staurosporine and lactacystin. Thanks to intensive, thorough and sustained work by researchers worldwide - not just by the scientists involved in their discovery - all of these compounds are continuing to reveal a wide variety of potentially useful but hitherto hidden and unrealised bioactive properties. The story of all three of these remarkable compounds is a successful and exemplary one, and each promises significant future advances and possible additions to our antibiotics armamentarium. More than anything, it confirms that to expedite the optimal discovery of new antibiotics we will need a comprehensive and well-coordinated programme to:

1. Identify new microbes
2. Improve cultivation techniques
3. Isolate as many new chemicals as we can from the microbes found
4. Optimally, test all the chemicals for any and all bioactive properties
5. Re-test all existing chemicals, wherever they may be stored, for hitherto unrecognised bioactive characteristics
6. Re-examine all stored microorganisms for previously overlooked secondary metabolite production

Initial steps are being taken in this regard, as demonstrated by the establishment of the Global Health Innovation Technology (GHIT) Fund, the Global Antibiotic Research and Development Partnership (GARDP) and CARB-X, among others. The 2018 discovery of a new antibiotic family (the malacidins), together with a novel polymyxin-based “immunobiotic” that could give old, non-effective antibiotics a new lease of life, indicates that progress is being made. However, a global effort with a centrally located mechanism to coordinate, analyse and inform the advances made by partners in academia, the private and public sectors (national, international and multilateral), as well as non-governmental organisations, funding and legal organisations, and affected communities will be needed if we are to realise the urgent need for new, novel, effective, acceptable and affordable antibiotics in the necessary timeframe.
